# A Method for Obtaining 3D Point Cloud Data by Combining 2D Image Segmentation and Depth Information of Pigs

**DOI:** 10.3390/ani13152472

**Published:** 2023-07-31

**Authors:** Shunli Wang, Honghua Jiang, Yongliang Qiao, Shuzhen Jiang

**Affiliations:** 1 College of Information Science and Engineering, Shandong Agricultural University, Tai’an 271018, China; 2021110531@sdau.edu.cn (S.W.); j_honghua@sdau.edu.cn (H.J.); 2 Australian Institute for Machine Learning (AIML), The University of Adelaide, Adelaide, SA 5005, Australia; 3 Key Laboratory of Efficient Utilisation of Non-Grain Feed Resources (Co-Construction by Ministry and Province), Ministry of Agriculture and Rural Affairs, Department of Animal Science and Technology, Shandong Agricultural University, Tai’an 271018, China; szjiang@sdau.edu.cn

**Keywords:** pig detection and segmentation, 3D point cloud, YOLOv5s, Res2Net bottleneck, precision livestock farming

## Abstract

**Simple Summary:**

This paper presents a technique for acquiring 3D point cloud data of pigs in precision animal husbandry. The method combines 2D detection frames and segmented region masks of pig images with depth information to improve the efficiency of acquiring 3D data. Our method achieves an average similarity of 95.3% compared to manually labelled 3D point cloud data. This method provides technical support for pig management, welfare assessment, and accurate weight estimation.

**Abstract:**

This paper proposes a method for automatic pig detection and segmentation using RGB-D data for precision livestock farming. The proposed method combines the enhanced YOLOv5s model with the Res2Net bottleneck structure, resulting in improved fine-grained feature extraction and ultimately enhancing the precision of pig detection and segmentation in 2D images. Additionally, the method facilitates the acquisition of 3D point cloud data of pigs in a simpler and more efficient way by using the pig mask obtained in 2D detection and segmentation and combining it with depth information. To evaluate the effectiveness of the proposed method, two datasets were constructed. The first dataset consists of 5400 images captured in various pig pens under diverse lighting conditions, while the second dataset was obtained from the UK. The experimental results demonstrated that the improved YOLOv5s_Res2Net achieved a mAP@0.5:0.95 of 89.6% and 84.8% for both pig detection and segmentation tasks on our dataset, while achieving a mAP@0.5:0.95 of 93.4% and 89.4% on the Edinburgh pig behaviour dataset. This approach provides valuable insights for improving pig management, conducting welfare assessments, and estimating weight accurately.

## 1. Introduction

Animal and livestock products are vital for human survival. However, the safety of the world’s food supply is endangered by factors such as coronaviruses, climate change, and dietary habits. As a result, the number of people experiencing global food shortages has increased significantly [1,2]. On the other hand, with the rise in living standards, people’s dietary structures are also changing. The level of consumption of meat and other protein-rich foods with integrity and high quality is rising [3,4]. According to data from the OECD-FAO Agricultural Outlook, pork is the most-produced livestock product in the world, accounting for nearly one-third of all meat consumption [5].

In recent decades, precision livestock farming technology has been widely used to promote normal pig production yield, improve farming production, save farming expenses, reduce feed wastage and environmental pollution, ensure food quality and safety, and benefit human health [6]. Monitoring the health status of pigs can help managers control the spread of diseases in time to protect healthy pigs [7]. Automatic or intelligent pig identification and monitoring are necessary for intensive pig farming and play a significant role in increasing the effectiveness of pig management and feeding [8]. Pig detection and segmentation, in particular, are the foundation for pig monitoring and continuously gathering data on pig welfare and health [9]. A reliable detection and segmentation technique can increase the accuracy of pig monitoring [10].

A variety of deep learning techniques for pig detection have been implemented as a result of the growth of convolutional neural networks (CNNs) [11,12]. Using Faster R-CNN, Yang et al. were able to localise pigs from cluster housing with an accuracy of 96.58% [13]. In 2020, Riekert et al. used the neural architecture search base network to improve Faster R-CNN’s backbone for extracting features from 2D images of pigs, resulting in an accuracy of 80.20% for model detection [14]. In 2021, Yin et al. improved YOLOv3 by adding an attention mechanism to complete real-time pig detection and achieved 94.12% detection accuracy [15]. Ahn et al. fused the YOLOv4 model that was trained with the original data and the YOLOv4 model that was trained with the enhanced data, which improved the detection performance of the model to 94.33% [16]. That same year, Wutke et al. established a CNN algorithm that followed the autoencoder structure to achieve 95.4% accuracy in pig detection [17]. In 2022, To tackle image noise triggered by factors such as flying insects, Bo et al. used a generative adversarial model to remove noise from images. Removing noise increased the algorithm’s accuracy from 76.6% to 90.6% [18]. Kim et al. achieved 99.44% detection accuracy and 30 frames per second real-time detection speed by embedding the lighter YOLOv4 model into an embedded circuit board [19]. Xiao et al. designed a cascaded Faster R-CNN pig detector to achieve 98.4% pig detection accuracy [20]. Highly accurate and efficient detection algorithms play an important role in smart farming.

In recent years, pig segmentation technology has also made considerable progress, and many researchers have proposed different methods to improve the accuracy and efficiency of segmentation. For example, In 2021, Tu et al. used Mask Scoring R-CNN (MS R-CNN) and the Soft NMS algorithm to achieve a new instance segmentation method [21]. Hu et al. proposed a new dual attention block and introduced it into the Feature Pyramid Network (FPN) to achieve instance segmentation performance with an average precision of 93.1% [22]. Zhai et al. used an improved Mask R-CNN to achieve effective segmentation of sticky pigs, with a segmentation accuracy of 91.7% [23]. In addition, in 2022, Liu et al. integrated DeepLab V3+, a model with an attention mechanism, and a model for high-frequency and low-frequency feature fusion to build an integrated segmentation model, achieving 76.31% MIoU [24]. Lu et al. used Swin Transformer method to achieve pig segmentation accuracy of 86.9% [25]. Gan et al. used an anchor-free network and a graph convolutional network to achieve piglet segmentation accuracy of 95.2% [26]. Zhong et al., based on living visible light and thermal infrared images, proposed a multi-source fusion algorithm, achieving a high-precision segmentation of pigs [27]. These methods have made considerable progress in addressing challenges such as complex backgrounds, variations in lighting conditions, and occlusion in pig segmentation, thereby offering valuable technical assistance for intelligent pig management and animal welfare assessment.

The 3D method is an extension of the 2D method and can solve some problems more effectively. Compared to the limitations of 2D methods in complex spatial environments and tasks, 3D methods can extract rich geometric and spatial information from three-dimensional data, providing more effective scale and location information for objects and more easily handling issues such as occlusion [28,29]. Based on deep learning, point cloud detection and segmentation tasks can be divided into two categories: indirect and direct point cloud segmentation [30,31]. Indirect point cloud detection and segmentation utilise CNN technology to process structured feature point cloud data, which requires preprocessing of point cloud data through gridding and voxelization. Many works use multiple-view 2D image reconstruction of 3D views [32,33,34] or 3D CNNs to achieve 3D detection and semantic segmentation tasks [35,36,37,38,39]. However, this method not only requires complex data preprocessing operations but also loses a large amount of point cloud information [30].

Direct point cloud segmentation abandons the complicated prior settings of point cloud data and directly utilises deep learning techniques for end-to-end learning of target point cloud features. In 2017, Qi et al. first proposed a point-level prediction network called PointNet, which can learn translation and rotation invariant information of unstructured point clouds [40]. Subsequent studies have focused on enhancing local region feature learning [41,42], graph-based point cloud neighborhood feature learning [43,44,45,46,47], and point cloud attention feature learning [30,48,49,50,51,52,53].

Three-dimensional point cloud detection and instance segmentation of pigs is a research direction with important application value. However, there are few studies on the detection and segmentation of pig 3D point clouds, and most focus on the estimation of pig weight. Some studies use devices such as Kinect V3 [54,55], Realsense [56,57,58], and a laser scanner [59] to collect point cloud data of single pigs, and remove background point clouds by setting the background depth to 0 and using the subtraction programme, achieving instance segmentation of pigs. In addition, Ref. [60] utilised an instance 2D segmentation network and depth image to perform accurate 3D point cloud segmentation for individual pigs, but they did not assess the effectiveness of the network in segmenting multiple pig 3D point clouds in complex environments. However, most of the existing methods are based on depth cameras to acquire point cloud data of individual pigs, and they utilise subtraction algorithms to remove ground point clouds for 3D detection and segmentation of single pigs [54,55,56,57,58,59]. These approaches not only encounter difficulties in removing extraneous object point clouds around the cleaning device but also struggle with the instance segmentation of multiple cleaning devices’ 3D point clouds to achieve an automatic estimation of the weights of all pigs in the pigsty.

Considering the challenges in 3D detection and segmentation to acquire 3D pig body information, we propose a novel approach that combines 2D detection and segmentation with depth information to enable easy and fast acquisition of 3D pig body point cloud information. The paper introduces a Res2Net block into YOLOv5 to enhance the model’s fine-grained feature extraction and improve its detection and segmentation performance. Additionally, the paper proposes a 3D point cloud detection and segmentation algorithm that uses depth data and generated detection and segmentation masks to achieve a simple and efficient 3D point cloud multi-pig detection and segmentation method.

This paper is structured as follows: Section 2 describes the dataset acquisition devices and schemes, as well as the improvement of the method. Section 3 introduces the experimental setup. Section 4 illustrates and discusses the experimental results of pig detection and segmentation. Section 5 discusses the advantages, limitations, and generalization of our approach. Finally, conclusions and recommendations for additional research are provided in Section 6.

## 2. Materials and Methods

### 2.1. Data Acquisition and Preprocessing

To validate the effectiveness of our approach in complex environments, we have constructed two datasets with complex environments (variations in lighting, etc.): our dataset and the Edinburgh (UK) pig behaviour dataset.

**Figure 1:**
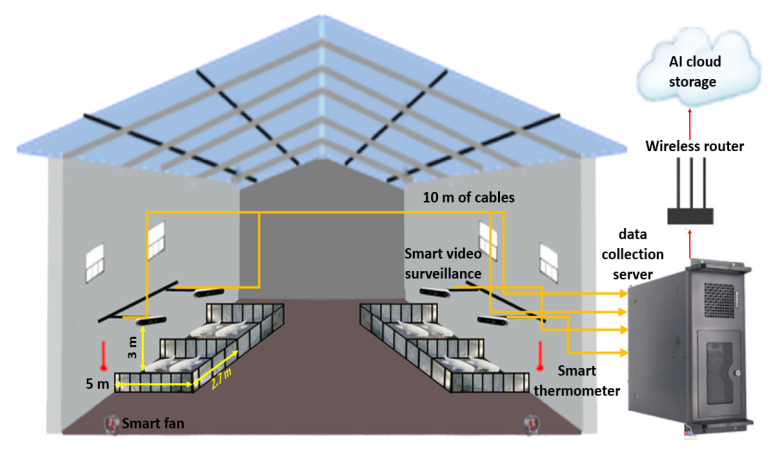
The whole data acquisition platform.

From 22 November 2021 to 10 January 2022, experimental data were gathered on a farm in Fanzhuang Village, Dongping County, Tai’an City, Shandong Province, China. On this farm, more than 150 New America Ternary pigs were bred in 16 pigsties. Each pigsty measured approximately 5 m in length and 2.7 m in width and was equipped with a drinking nipple and a feeding trough. Typically, each pen housed 7–10 pigs that were fed three times a day (morning, noon, and afternoon). Figure 1 depicts the entire data collection platform. Intel RealSense D455 depth cameras (Intel Corporation, Santa Clara, CA, USA) were mounted on a horizontal post 3 m high in the centre of each of four pigsties to capture top-view images of the entire pigsty. The resolution of the data collected by the D455 depth camera was set to 848 × 480 with an FPS of 15 and all other parameter configurations were set to their default values. The D455 used a 10-m Type-C connection to transmit the voluminous 3D point cloud data it gathered to the server.

**Figure 2:**
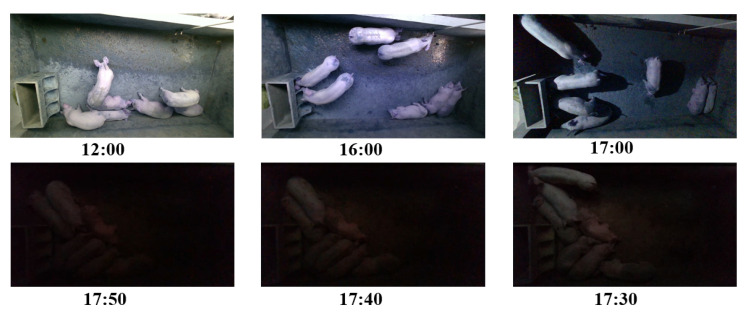
Acquired pig image examples in one day. The corresponding times are 09:00, 12:00, 16:00, 17:00, 17:20 and 17:30.

Changes in illumination present a significant challenge for long-term pig monitoring. To evaluate the effectiveness of the proposed approach, data were collected three times during the day (09:00, 12:00, and 16:00) and three times in the evening (17:00, 17:20, and 17:40) over several months as the pigs grew. A total of 2 h of data were collected each day, resulting in a total of 5400 daytime and 5400 nighttime image datasets. A selection of these images can be observed in Figure 2, which shows data acquired at 09:00, 12:00, 16:00, 17:00, 17:20, and 17:30.

**Figure 3:**
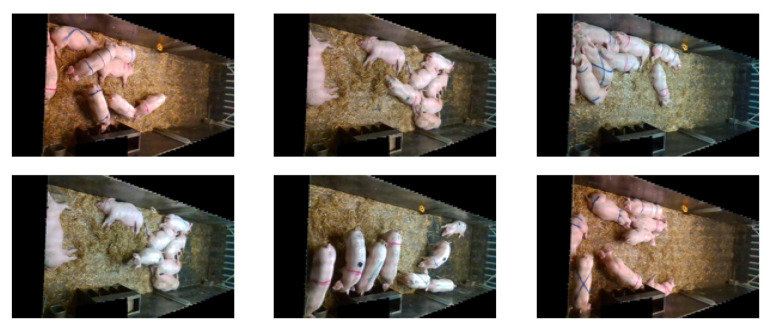
Edinburgh (UK) pig dataset.

**Figure 4:**
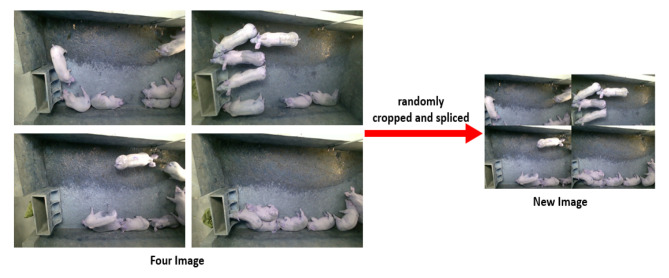
Data after mosaic data enhancement.

Although pig activity was more active during the data collection period, many redundant and similar images were still present in the collected data. To address this issue, 132,766 images in the PNG format were obtained by processing the original video stream data and extracting one image every 2 s. The double hash algorithm [61] was then used to identify and remove similar images, resulting in 5400 images after processing. We used a total of 6253 images from the Edinburgh dataset, as shown in Figure 3. The UK dataset is openly accessible at https://homepages.inf.ed.ac.uk/rbf/PIGDATA/ (accessed on 8–10 February 2021). To augment the training data, a mosaic data enhancement method was employed. This technique involves randomly cropping four images and stitching them together to create a new training image in Figure 4. In addition to enhancing the training data, this technique also increases batch size and improves the training effectiveness from different perspectives [62].

### 2.2. Fast 3D Detection of Pigs Based on YOLOv5s

**Figure 5:**
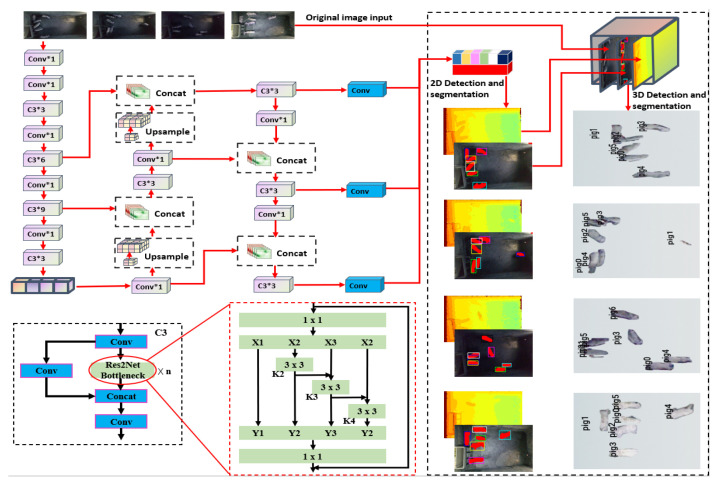
The fast 3D pig detection based on the improved YOLOv5s model structure.

Regular monitoring and recording of pig weight can provide valuable insights into their growth and development. Utilising 3D object detection technology to estimate the weight of pigs presents a superior approach compared to relying solely on 2D images. This technology can capture more comprehensive data on the body shape of pigs, resulting in more proficient and precise weight estimation. In this study, we implemented an enhanced version of the YOLOv5s model to facilitate efficient 3D pig detection and segmentation procedures using depth images, detection results, and semantic segmentation masks. We improved the YOLOv5s model’s performance in detection and segmentation by replacing its original bottleneck structure with a Res2Net bottleneck structure, which enhances the backbone network’s ability to extract fine-grained features. The fast 3D pig detection based on the improved YOLOv5s model structure is shown in Figure 5.

#### 2.2.1. Improved Backbone with Res2Net

The backbone’s ability to extract image features is critical to the overall network model. Gradient dispersion and feature redundancy are issues that YOLOv5s employs bottleneck modules to address throughout the feature extraction process. This paper introduced the Res2Net bottleneck in place of YOLOv5s’s bottleneck to enhance the backbone’s capability to withdraw features [63].

**Figure 6:**
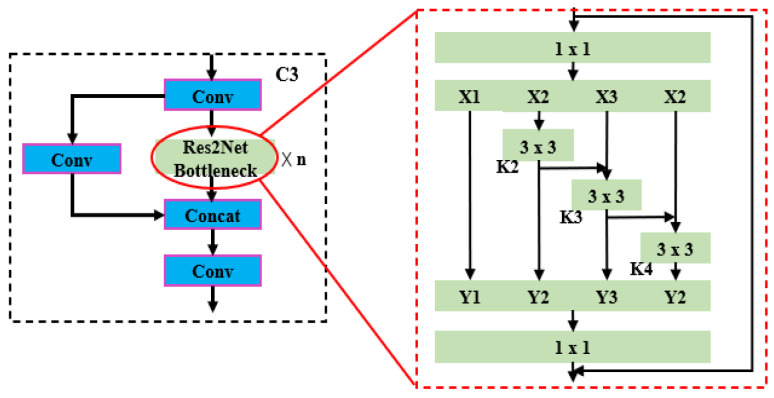
Res2Net bottleneck.

The Res2Net bottleneck builds hierarchical residual class connections inside the residual structure to extract multiscale features at the granularity level. As shown in Figure 6, this module performs residual concatenation by dividing convolutional features into four groups (X1, X2, X3, X4) and extracting features for each group in turn (K2, K3, K4). Finally, the four groups are concatenated into one group (Y1, Y2, Y3, Y4) to achieve finer-grained multiscale feature representation.

#### 2.2.2. Acquisition of 3D Information on Pigs

In pig feeding, the pig body size is important for describing pig growth and reproductive performance; the pig weight is important information for helping farmers optimise feed and management; and the pig body condition score is an important parameter for monitoring pig welfare and health. Compared with 2D vision technology, 3D vision technology is more advantageous for tasks such as body size measurement, body condition scoring, and weight prediction. Therefore, developing a fast and efficient multi-pig point cloud detection and segmentation technique is the key to achieving a real-time estimation of multi-pig weight.

To achieve these tasks, this study proposes a fast 3D pig detection and segmentation method based on 2D pig image detection and semantic segmentation. This method utilises the current mature 2D object detection and segmentation techniques, and can quickly complete 3D detection and segmentation without training, as shown in the pseudocode in Algorithm 1.    
**Algorithm 1:** Obtain the 3D point cloud coordinates of the detected object in the 2D image
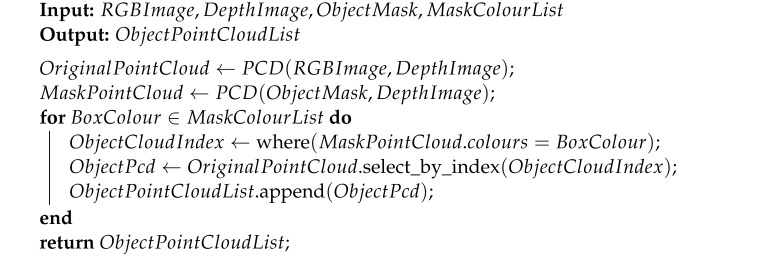



**Figure 7:**
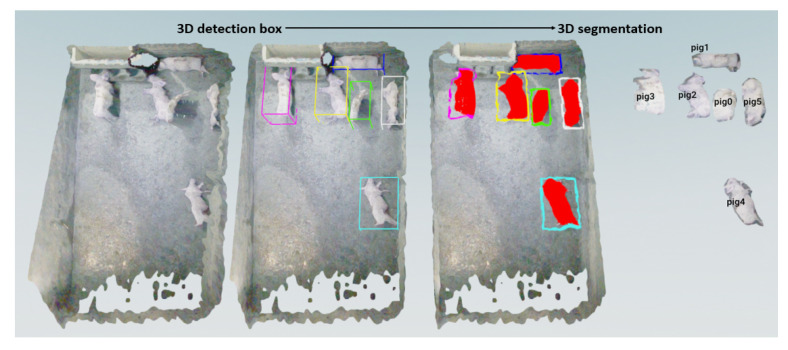
Three-dimensional point cloud multi-pig instance detection and segmentation. The coloured boxes represent different detection instances.

In Algorithm 1, RGBImage represents an array for reading the RGB images, DepthImage represents an array for reading the deep images, ObjectMask represents an array for reading the detection and segmentation mask images, MaskColourList represents the mask colours corresponding to different detection objects, and ObjectPointCloudList represents a list of point clouds for different objects. OriginalPointCloud refers to the point cloud that is generated from the RGB image and depth image, while MaskPointCloud represents the mask point cloud generated by the detection and segmentation mask image and depth image. We obtain the instance segmentation mask of pigs by combining detection frame masks and semantic segmentation masks, and we use the RGB image and depth image to achieve 3D point cloud multi-pig instance detection and segmentation in Figure 7.

## 3. Experimental Setup

### 3.1. Experimental Platform

In our experiments, a computer running Windows 10 Professional Edition (64-bit) with an Intel${}^{®}$ Core${}^{\mathrm{TM}}$ i7-11700 @ 2.50 GHz CPU (Intel Corporation) and NVIDIA GeForce GTX 1080Ti GPU (Nvidia Corporation, Santa Clara, CA, USA) was used (Table 1). The proposed method was programmed using the PyTorch v3.8 framework.

### 3.2. Experimental Dataset Construction

**Figure 8:**
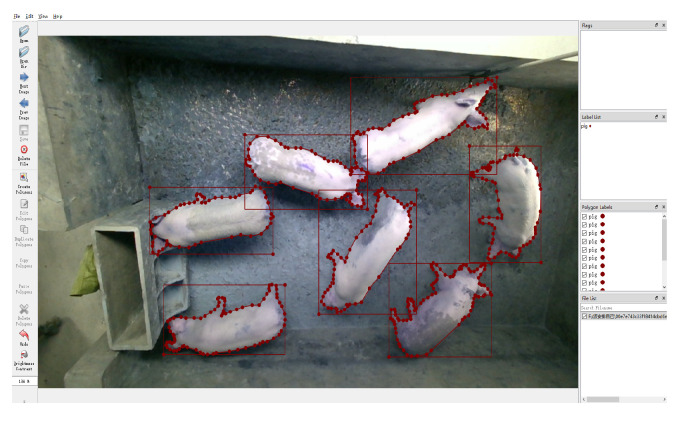
Pig segmentation labeling.

We utilised Labelme software [64] to perform detection and segmentation annotations on images of pigs (Figure 8), resulting in a dataset consisting of a total of 5400 images, with 2700 images for each category. One category contains images taken between 09:00 and 16:00, while the other category contains images taken between 17:00 and 18:00. For the dataset used in our experiments, 70% of the data were allocated to the training set and 30% to the testing set. In addition, we permuted the Edinburgh dataset in the same way to obtain a training set of 4377 images and a validation set of 1876 images.

### 3.3. Network Training Parameters

In this study, we set the parameters of these networks to be the same to enable a valid comparison between multiple models. As shown in Table 2, the learning rate was set to 0.04, the image size was set to 640 × 640, the batch size was set to 32, and the training epoch was set to 200. The optimizer used was a stochastic gradient descent. Additionally, YOLOv5s did not use pretrained weights to ensure a fair comparison between all models. The confidence threshold of the predicted boxes represents the probability that the model believes there is an object within that box. This paper uses the value of 0.9, and only when the confidence of a predicted box is above 0.9 is it considered an accepted and valid detection result. The hyperparameters used were based on the settings in the ‘hyp.scratch-low.yaml’ file in YOLOv5s, and all other parameters were set to their default values.

### 3.4. Comparison with State-of-the-Art Methods

In this study, we compared the improved method with state-of-the-art object detection models (SSD [65], Faster R-CNN [66], RetinaNet [67], Mask-RCNN [68], Fully convolutional networks (FCN) [69], and DeepLabv3 [70]). Faster R-CNN consists of a region proposal network and classification and regression networks and is considered the best model for the two-stage model. The SSD is composed of VGG16 [71] and a one-stage detector based on a feature pyramid. RetinaNet is composed of ResNet and a one-stage detector and employs FPN to increase the algorithm’s detection performance. Mask R-CNN extends Faster R-CNN and can perform both object detection and instance segmentation [68]. FCN is a network composed of fully convolutional layers, which can achieve end-to-end image segmentation [69]. DeepLabv3 is a network that uses techniques such as Atrous Spatial Pyramid Pooling to enhance segmentation accuracy [70]. The six models achieved more satisfactory results in object detection and were therefore selected and compared with our approach [65,66,67].

### 3.5. Performance Evaluation

The mean average precision (mAP) and confusion matrix were used independently to assess the effectiveness of the pig detection model. The confusion matrix is a standard format for representing the classification accuracy of a model and includes the number of identifications that were false-negative (FN), false-positive (FP), true negative (TN) or true positive (TP). TP indicates the number of pigs correctly predicted, FP indicates the number of pigs incorrectly predicted as the background, FN indicates the number of backgrounds incorrectly predicted as pigs, and TN indicates the number of backgrounds correctly predicted. Using the results of the confusion matrix are two evaluation measures, precision (Equation (1)). Precision represents the proportion of correctly identified pig pictures to all identified pig pictures.

(1) $$\mathrm{Precision}=\frac{\mathrm{TP}}{\mathrm{TP}+\mathrm{FP}}$$

In addition, mAP represents the mean average precision for each category (Equation (2)). We selected mAP@0.5 and mAP@0.5:0.95 evaluation metrics to more accurately reflect the model performance. The mAP value is indicated by mAP@0.5, and the average mAP at various IoU thresholds is indicated by mAP@0.5:0.95 (from 0.5 to 0.95). These accuracy metrics reflect the accuracy of pig and background differentiation in images from different angles. Global acc represents the proportion of correctly predicted pixels to the total number of pixels (Equation (3)). In this context, n represents the number of categories, and $p_{\mathrm{ij}}$ represents the element in the i-th row and j-th column of the confusion matrix, which indicates the number of pixels that are predicted as category j but truly belong to category i.

(2) $$\mathrm{mAP}=\frac{1}{n}\sum_{i=0}^{n}{\mathrm{AP}}_{i}$$

(3) $$\mathrm{GlobalACC}=\frac{\sum_{i=1}^{n}p_{\mathrm{ii}}}{\sum_{i=1}^{n}\sum_{j=1}^{n}p_{\mathrm{ij}}}$$

## 4. Results

### 4.1. Two-Dimensional Pig Image Detection and Segmentation Results

**Figure 9:**
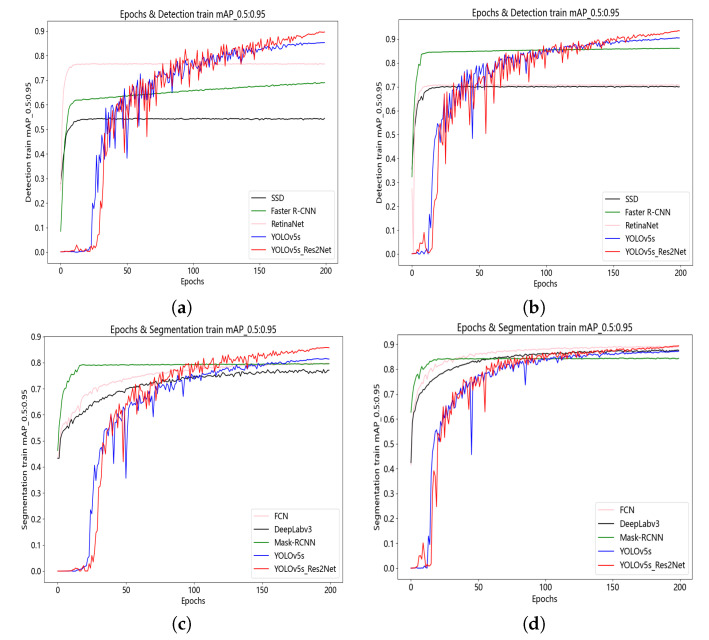
mAP@0.5:0.95 for the detection algorithm on our dataset (**a**). mAP@0.5:0.95 for the detection algorithm on UK dataset (**b**). mAP@0.5:0.95 for the segmentation algorithm on our dataset (**c**). mAP@0.5:0.95 for the segmentation algorithm on UK dataset (**d**).

We conducted a comparative analysis of six networks for detection and segmentation tasks, including three detection networks (SSD, Faster R-CNN, and RetinaNet) and three segmentation networks (FCN, DeepLabv3, and Mask RCNN). Figure 9a,b shows the training curves of different detection and segmentation models, respectively, on the mAP@0.5:0.95 metric for our dataset. Similarly, Figure 9c,d shows the training curves for different detection and segmentation models on the mAP@0.5:0.95 metric for the UK dataset.

From Figure 9a,b, we can observe that the improved YOLOv5s_Res2Net model outperforms YOLOv5s, SSD, Faster R-CNN, and RetinaNet on the mAP metric, achieving higher detection accuracy. In addition, although YOLOv5s_Res2Net fluctuates a lot in the early stages of training, it gradually decreases and stabilises in the later stages of training. From Figure 9c,d, we can observe that the improved YOLOv5s_Res2Net model outperforms YOLOv5s, FCN, DeepLabv3, and Mask RCNN on the mAP@0.5:0.95 metric, achieving higher segmentation accuracy. Similar to the YOLOv5s_Res2Net detection model, its segmentation model has a slow convergence speed and is unstable in the early stage of training, but gradually stabilizes in the later stage of training. In summary, the YOLOv5s_Res2Net model shows better performance in pig detection and segmentation tasks. Additionally, by analysing Figure 9a–d, we can observe that in detection and segmentation tasks on different datasets, the YOLOv5 algorithm consistently maintains a stable model performance compared to the other six algorithms. For example, in Figure 9a,b, the RetinaNet algorithm shows a large performance gap between two different datasets.

This experiment used the mAP metric to evaluate both detection and segmentation tasks. Table 3 shows the results of pig detection and segmentation on our dataset and the UK dataset using YOLOv5s_Res2Net, YOLOv5s, SSD, RetinaNet, Faster R-CNN, FCN, DeepLabv3, and Mask-RCNN methods. In our dataset, YOLOv5s_Res2Net achieved 99.5% and 99.5% mAP@0.5 for pig detection and segmentation tasks, respectively, and 89.6% and 84.8% mAP@0.5:0.95, representing an improvement of 4.3% and 3.2% mAP@0.5:0.95, respectively, compared to the YOLOv5s algorithm. In the UK dataset, YOLOv5s_Res2Net achieved 99.4% and 99.4% mAP@0.5 for pig detection and segmentation tasks, respectively, and 93.4% and 89.4% mAP@0.5:0.95, representing an improvement of 2.9% and 2.2% mAP@0.5:0.95, respectively, compared to the YOLOv5s algorithm. Moreover, we validated the effectiveness of the Mosaic algorithm on the UK dataset using YOLOv5s. The experimental results demonstrate that incorporating the Mosaic algorithm into YOLOv5s improved its detection and segmentation performance by 2.4% and 2.1% in terms of mAP@0.5:0.95, respectively.

**Figure 10:**
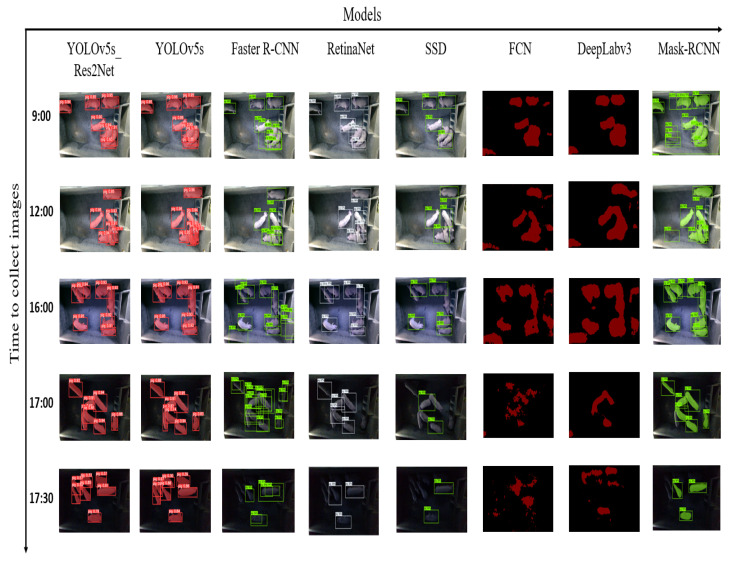
Detection results of improved YOLOv5s, Faster R-CNN, RetinaNet, SSD, FCN, DeepLabv3 and Mask-RCNN for images of the same pig at different times in the same pigsty. The different colours in the figure only represent the results generated by different models.

Additionally, Table 3 also shows the performance comparison of YOLOv5s_Res2Net with other algorithms on two tasks. For the detection task, in our dataset, YOLOv5s_Res2Net demonstrated an improvement of 1.0%, 14.8%, 3.6%, and 5.8% on the mAP@0.5 metric and 20.0%, 34.9 12.9%, and 16.6% on the mAP@0.5:0.95 metric compared to the Faster R-CNN, SSD, RetinaNet, and Mask-RCNN algorithms, respectively. In the UK dataset, YOLOv5s_Res2Net improved by 0.4%, 1.2%, 3.1%, and 3.7% on the mAP@0.5 metric and by 7.4%, 23.4%, 24.1%, and 9.3% on the mAP@0.5:0.95 metric compared to the Faster R-CNN, SSD, RetinaNet, and Mask-RCNN algorithms, respectively. For the segmentation task, in our dataset, YOLOv5s_Res2Net improved by 5.1%, 7.7%, and 6.8% on the mAP@0.5:0.95 metric compared to the FCN, DeepLabv3, and Mask-RCNN algorithms, respectively. In the UK dataset, YOLOv5s_Res2Net showed an improvement of 0.1%, 1.6%, and 7.1% on the mAP@0.5:0.95 metric compared to the FCN, DeepLabv3, and Mask-RCNN algorithms, respectively. It can be observed that YOLOv5s_Res2Net performs significantly better than other algorithms on both metrics. Figure 10 shows some pig images and their detection and segmentation results.

**Figure 11:**
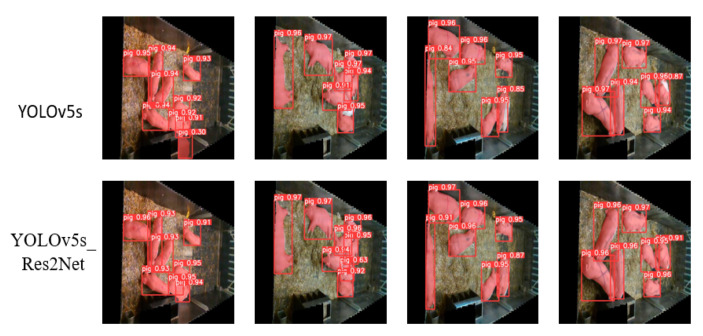
Detection segmentation plots of YOLOv5s and YOLOv5s_Res2Net on the UK dataset.

Figure 10 presents the detection and segmentation results of eight algorithms on data collected from five different time periods. From the figure, it can be observed that YOLOv5s_Res2Net accurately locates pigs. However, YOLOv5 produces more duplicate and incorrect detections when images are collected at 12 o’clock. Faster R-CNN has a high number of false detections, which negatively impacts the overall performance of the model. RetinaNet performs well on images with good lighting and no overlap but struggles with images that have poor lighting and overlap, resulting in missed and duplicate detections. The SSD algorithm outperforms both Faster R-CNN and RetinaNet in terms of detection performance. Figure 11 presents the detection and segmentation results of two algorithms, YOLOv5s and YOLOv5s_Res2Net, on the UK dataset. From the figure, it can be observed that YOLOv5s_Res2Net possesses more fine-grained image features compared to YOLOv5s, enabling better detection and segmentation of pigs.

In summary, YOLOv5s_Res2Net accurately identifies the location and outline of pigs. When compared to six other detection and segmentation algorithms, YOLOv5s_Res2Net demonstrates superior performance in detection and segmentation tasks under varying lighting conditions.

### 4.2. Three-Dimensional Point Cloud Based on Pig Segmentation Mask and Depth Information

**Figure 12:**
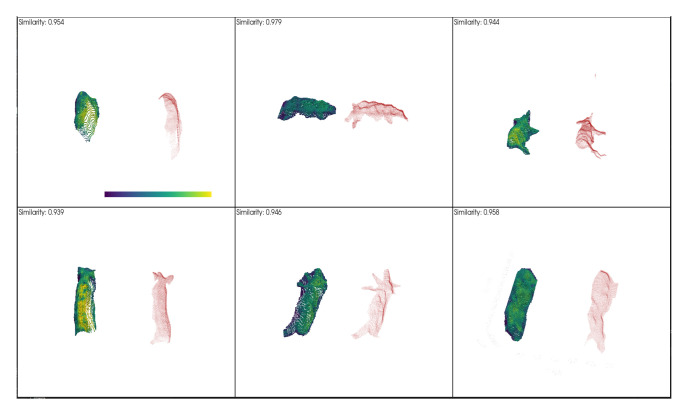
The comparison results between the 3D point clouds of pig segmentation (**left** and green) and the manually annotated point clouds (**right** and pink).

**Figure 13:**
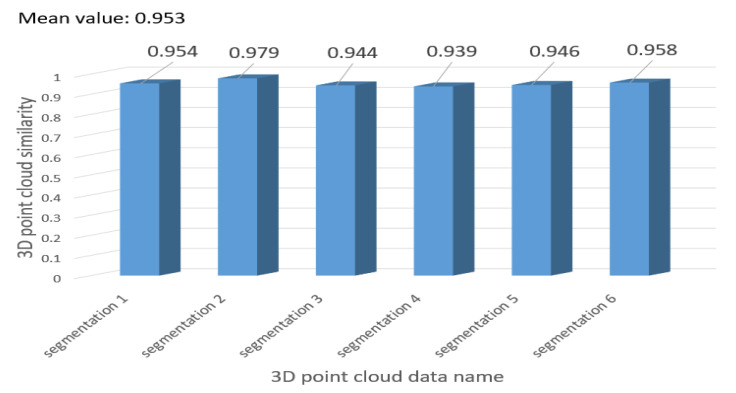
The similarity bar chart of our method in point cloud detection and segmentation.

We obtain 3D point clouds of pigs by 2D detection and segmentation. To assess the consistency between our method and manual annotation, we applied the ICP algorithm to align the two types of point clouds and computed their similarity scores. Figure 12 displays the comparison results between the 3D point clouds generated by our pig segmentation method (left) and the manually annotated point clouds (right). Figure 13 presents a bar chart depicting the similarity between our method and the manually annotated point cloud, with an average similarity of 95.3%. These results indicate that our method is highly accurate and reliable.

## 5. Discussion

### 5.1. Advantages and Limitations

Our method offers several advantages: (1) It employs a single YOLOv5 model to perform both detection and segmentation tasks. (2) It leverages mature 2D image technology to rapidly complete 3D point cloud detection and segmentation tasks. (3) The training difficulty and detection speed of our 3D point cloud detection and segmentation method are comparable to those of a 2D detection and segmentation model. (4) Our method effectively avoids the generation of redundant point clouds due to human errors.

**Figure 14:**
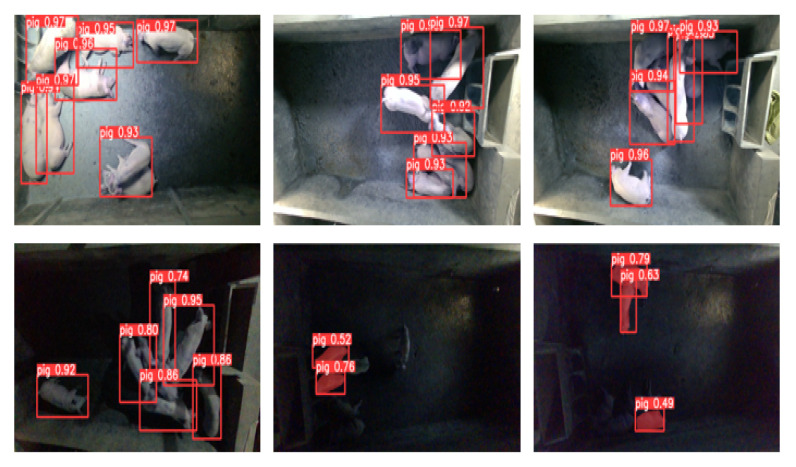
Unsuccessful detection and segmentation of pigs.

However, our method relies on 2D detection results, which means that the effectiveness of point cloud detection and segmentation is limited by the performance of the 2D detection task. As illustrated in Figure 14, when there is excessive adhesion of the target, the performance of the detection and segmentation models will decrease.

### 5.2. Generalization of the Model

**Figure 15:**
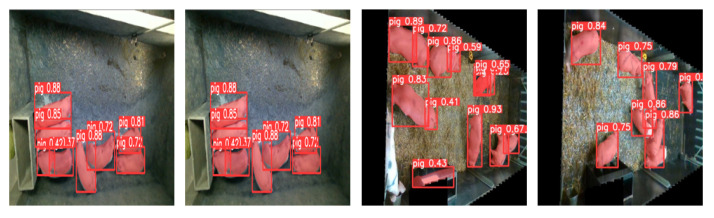
Models trained on different datasets recognise additional dataset’s images.

Considering the potential applications of this research in the pig farming industry, we tested models trained on different datasets on another dataset. Figure 15 shows the detection and segmentation plots of the two models on our dataset and the UK dataset, respectively. From the image, we can observe that models trained on different datasets have some issues recognising each other’s images. These issues include incomplete segmentation of pigs’ edges, inaccurate identification of incomplete pig targets, and misidentification of other objects in the pigsty as pigs. These problems indicate that our method’s generalisation ability is not strong enough to recognise pig images with backgrounds of cement floors and grass. However, we believe that with a sufficient number of training samples, the generalisation ability of our model can be effectively improved, enabling our method to be applicable to datasets with different backgrounds and improving the accuracy of pig recognition in various backgrounds.

## 6. Conclusions

This paper proposes a detection and segmentation algorithm that is based on YOLOv5s. The algorithm enhances the fine-grained feature extraction ability of the backbone network by using a Res2Net bottleneck on the basis of YOLOv5s and significantly improves the detection and segmentation performance of the model in complex environments (variations in lighting). The experimental results demonstrated that the improved YOLOv5s_Res2Net achieved a mAP@0.5 of 99.5% for both pig detection and segmentation tasks on our dataset, while achieving a mAP@0.5:0.95 of 89.6% and 84.8%, respectively. On the Edinburgh pig behaviour dataset, YOLOv5s_Res2Net obtained a mAP@0.5 of 99.4% for both tasks, along with a mAP@0.5:0.95 of 93.4% and 89.4%. In comparison, YOLOv5s_Res2Net outperformed Faster R-CNN, SSD, RetinaNet, FCN, DeepLabv3, and Mask-RCNN algorithms.

In addition, we obtained detection and segmentation masks for RGB-D data using 2D detection and segmentation algorithms to obtain 3D point cloud data of multiple pigs simply and efficiently, with an average similarity of 95.3% compared to manually labelled point cloud data. This method uses mature 2D computer vision techniques, avoiding the tedious task of 3D data annotation and the high-performance hardware required for deep learning training based on 3D data, and can quickly and efficiently analyze the 3D point cloud data of pigs. In future research, we will implement point cloud reconstruction and weight estimation of multiple pigs based on this algorithm to provide technical support for intelligent farming.

## Figures and Tables

**Table 1:** The computer’s experimental equipment.

Hardware	Type
CPU	11th Gen Intel${}^{®}$ Core${}^{\mathrm{TM}}$ i7-11700 @ 2.50 GHz
GPU	NVIDIA GeForce GTX 1080Ti
Memory	16.0 GB
Video Memory	11.0 GB
Hard disk	4 TB

**Table 2:** Training parameters for the models.

Parameters	Value	Remarks
Image size	640	Shrank image size to 640 × 640 during training.
Batch size	32	Ensured maximum batch size with limited graphics card memory.
Training epochs	100	Ensured that network was well-trained.
Optimiser	SGD	Selected suitable optimiser with limited graphics card memory.
Learning rate	0.04	Learning rate during model training.
Initial weights	Random	No pretraining weights were used.
Predict_conf_thres	0.9	The confidence threshold of predict boxse.
Hyperparameter	hyp.scratch-low.yaml	Hyperparameter file in YOLOv5s.

**Table 3:** Comparison of different detection methods (%).

Authors, Year	Methods	Datasets	mAP@ 0.5-D	mAP@ 0.5:0.95-D	mAP@ 0.5-S	mAP@ 0.5:0.95-S	Global ACC
Ren et al., 2015 [66]	Faster R-CNN	Our dataset	94.5	69.0	✔	✔	✔
Liu et al., 2016 [65]	SSD	Our dataset	84.7	54.7	✔	✔	✔
Lin et al., 2017 [67]	RetinaNet	Our dataset	95.6	76.7	✔	✔	✔
Ren et al., 2015 [66]	Faster R-CNN	UK dataset	99.0	86.0	✔	✔	✔
Liu et al., 2016 [65]	SSD	UK dataset	98.2	70.0	✔	✔	✔
Lin et al., 2017 [67]	RetinaNet	UK dataset	96.3	70.7	✔	✔	✔
Long et al., 2015 [69]	FCN	Our dataset	✔	✔	✔	79.7	94.6
Chen et al., 2017 [70]	DeepLabv3	Our dataset	✔	✔	✔	77.1	93.0
He et al., 2017 [68]	Mask-RCNN	Our dataset	93.7	73.0	92.6	78.0	✔
Long et al., 2015 [69]	FCN	UK dataset	✔	✔	✔	89.3	97.1
Chen et al., 2017 [70]	DeepLabv3	UK dataset	✔	✔	✔	87.8	96.2
He et al., 2017 [68]	Mask-RCNN	UK dataset	95.7	84.1	93.3	82.3	✔
Jocher et al., 2022 [62]	YOLOv5s	Our dataset	99.4	85.3	99.4	81.6	✔
Our work	YOLOv5s_Res2Net	Our dataset	99.5	89.6	99.5	84.8	✔
Jocher et al., 2022 [62]	YOLOv5s	UK dataset	99.3	90.5	99.3	87.2	✔
Our work	YOLOv5s_Res2Net	UK dataset	99.4	93.4	99.4	89.4	✔

## Data Availability

The authors do not have permission to share data. The code implementation for this article link is: https://github.com/Shunli-W/2D-3D-D-S.git, 7 June 2023.
